# Deep skin diseases diagnostic system with Dual-channel Image and Extracted Text

**DOI:** 10.3389/frai.2023.1213620

**Published:** 2023-10-19

**Authors:** Huanyu Li, Peng Zhang, Zikun Wei, Tian Qian, Yiqi Tang, Kun Hu, Xianqiong Huang, Xinxin Xia, Yishuang Zhang, Haixing Cheng, Fubing Yu, Wenjia Zhang, Kena Dan, Xuan Liu, Shujun Ye, Guangqiao He, Xia Jiang, Liwei Liu, Yukun Fan, Tingting Song, Guomin Zhou, Ziyi Wang, Daojun Zhang, Junwei Lv

**Affiliations:** ^1^The Third Affiliated Hospital of Chongqing Medical University (CQMU), Chongqing, China; ^2^Shanghai Botanee Bio-technology AI Lab, Shanghai, China; ^3^School of Medicine, Shanghai University, Shanghai, China; ^4^Department of Dermatology, Army Medical Center, Chongqing, China; ^5^School of Public Health, Li Ka Shing Faculty of Medicine, The University of Hong Kong, Pokfulam, Hong Kong SAR, China; ^6^School of Pharmacy, East China University of Science and Technology, Shanghai, China; ^7^Faculty of Science, The University of Sydney, Sydney, NSW, Australia; ^8^Faculty of Science, The University of Melbourne, Parkville, VIC, Australia; ^9^Chongqing Shapingba District People's Hospital, Chongqing, China; ^10^Shanghai Medical College, Fudan University, Shanghai, China; ^11^Huazhong Agricultural University, Wuhan, Hubei, China

**Keywords:** artificial intelligence, computer vision, skin disease, dermatitis, digital medicine

## Abstract

**Background:**

Due to the lower reliability of laboratory tests, skin diseases are more suitable for diagnosis with AI models. There are limited AI dermatology diagnostic models combining images and text; few of these are for Asian populations, and few cover the most common types of diseases.

**Methods:**

Leveraging a dataset sourced from Asia comprising over 200,000 images and 220,000 medical records, we explored a deep learning-based system for Dual-channel images and extracted text for the diagnosis of skin diseases model DIET-AI to diagnose 31 skin diseases, which covers the majority of common skin diseases. From 1 September to 1 December 2021, we prospectively collected images from 6,043 cases and medical records from 15 hospitals in seven provinces in China. Then the performance of DIET-AI was compared with that of six doctors of different seniorities in the clinical dataset.

**Results:**

The average performance of DIET-AI in 31 diseases was not less than that of all the doctors of different seniorities. By comparing the area under the curve, sensitivity, and specificity, we demonstrate that the DIET-AI model is effective in clinical scenarios. In addition, medical records affect the performance of DIET-AI and physicians to varying degrees.

**Conclusion:**

This is the largest dermatological dataset for the Chinese demographic. For the first time, we built a Dual-channel image classification model on a non-cancer dermatitis dataset with both images and medical records and achieved comparable diagnostic performance to senior doctors about common skin diseases. It provides references for exploring the feasibility and performance evaluation of DIET-AI in clinical use afterward.

## 1. Introduction

In China, 240 million people visit dermatologists annually, which accounts for 3% of all medical visits (Zhou et al., 2015). The number of dermatologists in China shows a significant imbalance. In China's public hospitals, there are approximately 29,800 dermatologists, but there are only 2.1 dermatologists for every 100,000 people. The number of dermatologists is markedly insufficient compared to the recommended standard of 4 dermatologists per 100,000 individuals. As of 2022, the number of practicing dermatologists and medical cosmetologists (and assistants) has only increased by 0.2%, which has exacerbated the shortage of dermatologists. However, in many areas, skin disease cases are diagnosed only by general practitioners. According to previous research, non-specialist diagnosis is obviously not able to meet the need for disease diagnosis and is clinically only 24–70% accurate (Federman and Kirsner, 1997; Federman et al., 1999; Tran et al., 2005; Moreno et al., 2007). Further, technological inequality aggravates the unevenness among doctors and medical centers. With recent technological developments, skin imaging equipment is applied in many top hospitals, which has enhanced the proficiency of dermatologists. However, the skin imaging system equipment is still insufficient and distributed unevenly, particularly in remote areas and primary hospitals in economically undeveloped areas (Li et al., 2021). Similarly, there are significant regional imbalances in the level of relevant clinical research and application, which further widen the gap in the diagnostic performance of physicians of different seniority and reveal that physicians utilize professional skin imaging equipment and intelligent auxiliary diagnosis tools for skin diseases to improve their diagnostic performance. Physicians can enhance the performance of dermatological diagnosis with the help of professional skin imaging equipment and improve disease awareness with dermatological assistant diagnostic tools, thereby increasing their diagnostic level. Through a cross-sectional study, most dermatologists are willing to receive the assistance of artificial intelligence tools to ensure time efficiency, diagnostic accuracy, and strengthening of patient management. Therefore, a novel tool for skin disease diagnosis and assessment will empower primary dermatologists and general practitioners with the diagnostic experience of leading dermatologists (Jain et al., 2021). This tool should achieve tiered diagnosis and treatment of dermatology and increase the diagnostic accuracy of primary dermatologists, thereby improving the diagnostic quality of dermatology in China.

Dermatology, as a discipline that largely relies on morphological characteristics for diagnosis, often depends on meticulous observation of visual patterns and disease classification through image feature extraction. To enhance the accuracy of skin disease image classification, Cascinelli et al. (1987) highlighted the importance of extracting features from dermoscopy images and used machine learning methods like the K-Nearest Neighbors (KNN) classifier to differentiate lesions, preliminarily distinguishing malignant from benign changes. For further optimization, She et al. (2007) suggested incorporating the traditional ABCD rule (Asymmetry, irregular Borders, Color variation, and Diameter) during the machine learning classification process, enhancing the precision of image categorization. Aiming to improve the classification of multi-source images, Barata et al. (2014) integrated a color consistency algorithm, elevating the diagnostic sensitivity and specificity. Additionally, AI models can alleviate physicians' workloads, assisting them with disease diagnosis. Premaladha and Ravichandran (2016) showcased the significant advantages of deep learning models in skin disease diagnosis compared to traditional diagnostic methods. Premaladha introduced a deep learning approach combined with SVM, which aids dermatologists in making diagnoses more accurately and in reducing unnecessary biopsies. Esteva et al. (2017) fully leveraged the capabilities of Deep Convolutional Neural Networks (CNN), demonstrating the model's efficiency in segmenting skin diseases, with its performance even being comparable to that of trained dermatologists. Albahar (2019) and Kassem et al. (2020) further delved into the deep learning application in distinguishing between benign and malignant lesions. Albahar (2019) incorporated a new regularizer into deep CNN learning, achieving high differentiation between various skin cancer malignancies and benign lesions. Kassem et al. (2020) emphasized the advantages of using pre-trained models and transfer learning techniques. However, technological progress comes with challenges. The size and the coverage of the dataset matter. Veronese et al. (2021) highlights the challenge of device limitations and the limited size of the dataset. Meanwhile, Haggenmüller et al. (2021) pointed out that most previous models were trained and tested on single images, while their test sets did not represent the real-world application comprehensively. The majority of earlier research has concentrated on distinguishing between benign and malignant lesions, an instance of which is Stanford University's work on the deep learning diagnosis of skin cancer in 2017 (Lu et al., 2012; Esteva et al., 2017; Seité et al., 2019). In summary, as described by Goyal et al. (2020), although AI systems have shown a high performance and substantial potential in skin disease diagnosis, they are still in the early stage of clinical application with many challenges ahead. Thus, our research aims to alleviate the diagnostic pressure on dermatologists and further optimize the diagnostic efficacy of AI models.

There are three benefits to applying deep convolutional neural networks to skin diseases. Firstly, data collection is easy because of the high incidence rate of skin diseases. Secondly, for skin diseases, the information is noisy and multimodal with a complicated context, which is better handled by deep networks. Thirdly, most cases of clinical skin disease diagnosis are conducted under natural light, which shares a similar distribution with large-scale image datasets like ImageNet, upon which we pretrained our image model. Among the algorithmic applications in dermatology, AI is mainly applied to computer vision algorithms. The academics of Stanford University trained the model using a database with 129,450 clinical images involving 2,032 diseases. AI equally matched 21 dermatologists in two tests, demonstrating that it can accurately classify skin cancer (Esteva et al., 2017). Haenssle et al. (2018) published a study assessing the ability to differentiate benign and malignant dermatology, comparing the diagnostic result of the deep learning model with 58 dermatologists, which showed that deep learning algorithms have higher specificity than dermatological specialists. The area under the curve (AUC) of ROC was greater than that of the dermatological specialists (0.86 and 0.79, respectively, *p* < 0.01), with most specialists underperforming compared to the deep learning model. However, most of the clinical research focused on the identification of benign and malignant dermatological disorders. The articles on diagnosing other diseases are insufficient and do not entirely cover the scenario of dermatological diagnosis. In 2020, Google Health trained the deep learning model with 64,837 images from 16,114 cases and verified the model with 14,883 images from 3,756 cases to learn and identify 26 common skin diseases. It compared the diagnostic results with 18 clinicians, including dermatologists, primary care physicians, and nurse practitioners. The diagnostic performance of the model was no worse than that of dermatologists and higher than that of six primary care physicians and six nurse practitioners. In Asia, there is a high burden of skin disease (Urban et al., 2021), while the level of AI model application in Asia is still lagging behind. This could lead to three problems. Firstly, the incidence rate of malignant melanoma in China is relatively low. According to the “2022 Cancer Facts & Data,” the United States has over 5 million new cases of skin cancer annually, with the melanoma incidence rate for white non-Hispanic people being 30 times that of other ethnicities. Nonetheless, much current academic research and challenges from the ISIC database mainly focus on the identification of benign and malignant skin tumors (Kleinberg et al., 2022), which means they do not satisfy the requirements of the Chinese ethnic group. Secondly, China and other countries experience a high incidence of skin diseases. These pronounced regional and ethnic differences underscore the importance of optimizing AI diagnostic models for specific populations. Thirdly, research indicates that current datasets used for AI diagnosis of skin diseases are noticeably biased toward certain populations, lacking comprehensive coverage across various skin conditions, like tones and roughness, among different ethnicities. Of the 70 reviewed studies, only 14 described the racial or ethnic information of the patients involved in the datasets, and only seven articles addressed skin color data. The majority of images are predominantly of individuals with light skin tones. Such dataset biases may undermine the accuracy of AI models when diagnosing skin diseases in Asian populations. Thus, it is critical to develop an AI model with a wide enough range of dermatological diseases for Asian populations (Health, 2020). Xiangya-Derm, the Chinese dermatology database established in 2020, classified six categories of common diseases and achieved an accuracy of 84.77% of the top 3, which is higher than the average accuracy rate of dermatologists (78.15%) (Huang et al., 2021). Thus, it is critical to develop an AI model with a wide enough range of dermatological diseases and sufficiently accurate diagnoses to be applicable to Asian populations (Health, 2020). Xiangya-Derm, the Chinese dermatology database established in 2020, classified six categories of common diseases and achieved an accuracy of 84.77% of the top 3, which is higher than the average rate of dermatologists (78.15%) 28 (Huang et al., 2021). However, the number of training and testing sets are 2,400 and 600, respectively (the ratio of training size: testing size is 4:1). For example, adding more research on typical cases of common outpatients on the basis of six corresponding representative skin diseases will lay a good foundation for the development of artificial intelligence skin disease diagnosis model. The Global Burden of Disease divides skin and subcutaneous tissue diseases into 12 categories (Roth et al., 2018; Vos et al., 2020), with more than 3,000 skin diseases. The medical data cannot be unified, the diagnosis and treatment data dimension in the field of dermatology is significantly smaller than in other medical fields, and it is obvious that AI models covering more kinds of dermatology are more positive for clinical diagnosis. Notably, the AI model covering more dermatological types is more positive and significant for clinical diagnosis. Thus, we selected 31 of the most common skin diseases and classified them into epidermal inflammatory skin diseases, dermal inflammatory skin diseases, and non-inflammatory skin diseases depending on dermatopathology (Ackerman et al., 2005). In addition, diagnosis based on information from images alone is not entirely reliable. During the process of diagnosis, the doctor needs multimodal information, especially medical records, for auxiliary diagnosis. Although many papers showed that the performance of AI in skin disease diagnosis is better than that of doctors, it is unfair to compare this performance with that of doctors in a scenario solely based on images, and it also does not reflect the real-world situation in a clinic. Haggenmüller et al. (2021) showed that “there is a need for truly prospective studies comparing the clinicians' diagnoses after real-life face-to-face patient examinations with the results of AI-based classification models.” Additionally, the use of AI models in dermatological diagnosis needs to show definitive efficacy in clinical trials or in the real world, and interventions involving AI need to undergo a rigorous prospective evaluation to demonstrate their impact on health outcomes before they can move to the next stage of clinical implementation (Rivera et al., 2020).

Based on the above, we are making an effort to find a solution. To begin with, we conducted a multicenter clinical study from September 2021 to December 2021, recruiting a total of 6,043 patients with dermatological diseases prospectively. To further clarify and explore the accuracy of a DIET-AI diagnosis, six doctors of different seniority were recruited for comparison, and they did not participate in our prospective data collection. Both DIET-AI and the dermatologist were enrolled in two diagnostic experiments: the image group and the image with medical records group. By evaluating the performance of doctors and DIET-AI, we found that the latter was more robust and not inferior to the senior, intermediate, and junior doctors we recruited. Next, we will further validate the potential of DIET-AI to assist general practitioners and junior dermatologists in diagnosing skin conditions in different clinical scenarios.

## 2. Methods

**Figure 1:**
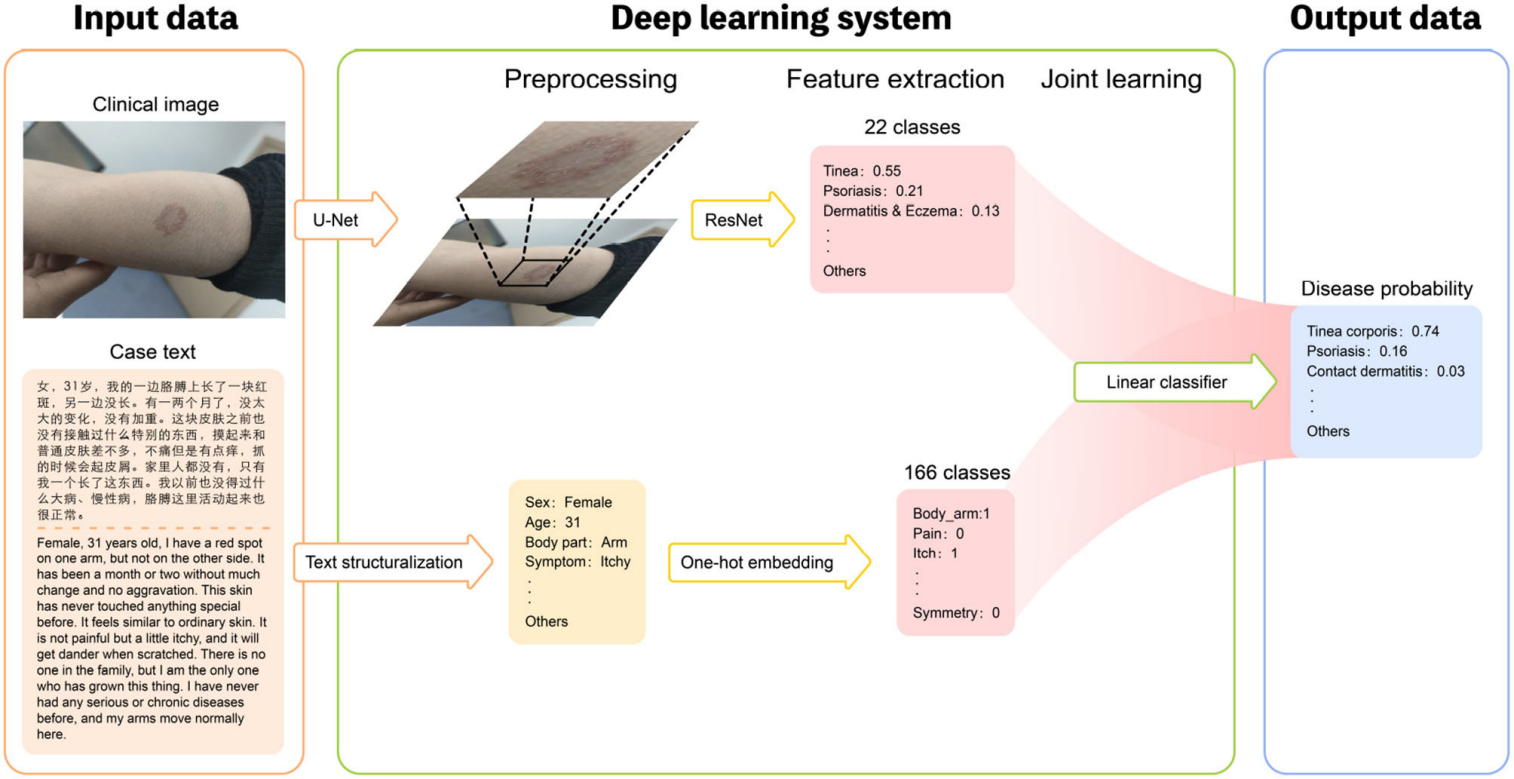
Overview of the structure of our DIET-AI model. For each case, the clinical images of the lesion site and case text of the medical record (including basic demographic information, lesion characteristics, medical history, etc.) were required as input data **(left)**. The Deep learning system includes three steps: preprocessing, feature extraction, and joint learning **(middle)**. In the preprocessing part, the clinical images will locate the bounding box of possible skin lesions through the U-Net network, and case text identifies text information through text structurization. In the feature extraction process, the main output of the image layer through the ResNet network is the relative probability of the main skin disease of 22 categories, and the main output of the text layer through the One-hot embedding is the relative probability of the relative probability of skin state of the 166 categories. The models are then connected through joint learning to form a complete model. Thus, the disease probabilities of 31 categories are mainly output as output data **(right)**. In this way, the DIET-AI model completes the combination of skin disease clinical images and medical case text information to provide a differential diagnosis for skin disease prediction.

To generate the predictive result, we build a joint classification model based on Dual-channel Image and Extracted Text features (DIET). The architecture consists of three separate components: (1) the image encoder consists of a detection model implemented with CenterNet and a classifier implemented with ResNet, generating the confidence value of the current prediction; (2) an NER model as the text encoder, and generate the one-hot vectors as our text feature; (3) a logistic regression model combining the text feature and image feature that gives the final prediction. The procedure is as follows: to train the detection model, we first crop the image of lesions from the original image in the original medical dataset so that we are able to form a training set without the background noise. Then, we train the image encoder with ResNet, calibrated with Platt Scaling, attaining the image feature. At the same time, the NER model will extract the keywords with medical meaning from the patient's medical records. The text feature is a one-hot vector that each element represent if the corresponding name entity are mentioned. With the confidence vector as our image feature and one-hot keyword vector as our text feature, we can then use a linear classifier, to be more specific, logistic regression, to give the prediction. The outline of this algorithm can be found in Figure 1. We will discuss each component in the following part.

For the detection model, we inherit the CenterNet (Zhou et al., 2019) architecture. As an anchor-free model, the object is represented by the center of its bounding box in this model. Afterward, the detector uses keypoint estimation to find center points. The model is optimized by minimizing the following Loss function:


$$L_{det}=L_{K}+\lambda_{size}L_{size}+\lambda_{off}L_{off}$$

where the *L*_*det*_ means the total loss function of the detection model. *L*_*K*_ is the focal loss, which estimates how much area the predicted window overlapped with the ground truth, *L*_*size*_ is a penalty function for the size of the predicted window, *L*_*off*_ represents the distance between the offset of the predicted window and the offset of the ground truth window. λ_*size*_ and λ_*off*_ are control parameters. In our work, we set λ_*size*_ to be 0.1 and λ_*off*_ to be 1. This model locates the bounding boxes of all possible lesions. In our work, it helps us to crop the image of lesions from the original environment.

For the image classifier, we inherit the ResNet (He et al., 2016) architecture to generate the initial probability distribution of the diseases. In the previous neural network structures, as the network depth increases, the accuracy soon achieves a degrades rapidly, which is called degradation problem. The ResNet architecture views the layers as learning residual functions with reference to the layer inputs. In this structure, the shallow layers build shortcut connections to higher layers and thus mitigate the degradation problem. In one following work (Li et al., 2017), Resnet is also demonstrated to have a smoother loss landscape than an MLP with the same number of parameter layers. We use both the original image and the cropped image as the input. By concatenating the final layer of both images, the Softmax layer will generate the model's prediction. Thus, we use image-only data to train a classification model. The loss function is a typical cross-entropy loss:


$$L=-\overset{N}{\underset{i=1}{\sum }}\overset{M}{\underset{j=1}{\sum }}\left[y_{ij}\log{ŷ}_{ij}\right]$$

where *N* is the number of the cases and *M* is the number of diseases. *y*^*i*^ means a ground truth vector. For each element in that vector, if the patient has the disease *j*, then *y*^*ij*^ = 1; otherwise, *y*^*ij*^ = 0. ŷ^*i*^ means the prediction vector that the ResNet made, the element of which is a number in the range of [0, 1]. This loss function means that the closer the model's prediction is to the ground truth, the less loss there will be.

Given its high prediction performance, the ResNet model may lead to bias when estimating the confidence. Guo et al. (2017) shows that the output of ResNet does not always reflect its ground truth correctness likelihood. As they suggested, to calibrate the model output, the Platt scaling method can be applied after the neural network inference. Platt scaling is a method that further trained a logistic regression layer from the output of our ResNet model in order to maximize the classification accuracy on a split dataset for calibration. We split 1/10 of the original valid dataset as our calibration dataset. Since the output of the logistic regression model is a probability by definition, this model is expected to be more calibrated.

Bare image information may not be enough since symptoms like itching or pain cannot be read directly from the image. Therefore, to incorporate expert knowledge and patient description, text mining on the EHR (Electronic Health Records) can be a feasible way. To extract all the name entities from the EHR, we use a Named Entity Recognition (NER) model, which is called the FLAT (Flat-LAttice Transformer) (Li et al., 2020) model. The filtered name entities then are reviewed by human to find the potential symptoms that helpful for the diagnosis. By combining these paired images and its symptoms, we further train a model that significantly improve the model performance. Their code is available on Github.^1^

Finally, by combining these paired image features and text features, we further trained a logistic regression model for prediction. This significantly improved the performance compared to a model that used only images. Although we experimented with other classifiers like single-layer neural networks, linear SVM, and xgboost, none performed as well as the logistic regression model. The logistic regression model can be seen as a one layer neural network for binary classification, which shares a similar loss to the ResNet:


$$L=\overset{N}{\underset{i=1}{\sum }}\left[y_{i}log{ŷ}_{i}+\left(1-y_{i}\right)log\left(1-{ŷ}_{i}\right)\right]$$

where *N* means the number of cases. *y*_*i*_ is the ground truth number, where this is 1 for the correct category and vice versa. ŷ_*i*_ is the prediction probability. As the logistic regression is for binary classification, we applied the model to the multi-classification problem by the One-vs-All method. In this method, we split the original multi-class classification into one binary classification problem per class. The final output will be the probability of each disease for the patient.

It is worth noting that our image encoder and text encoder are trained separately. The reason is that the triple data (disease-image-text) are rare compared to image-only data or text-only data. By separately training the image encoder (ResNet) and text encoder (FLAT), and jointly training the final classifier, we successfully mitigate the influence of the scarcity of the triple data.

## 3. Dataset

All image data were collected from patients at every health center. After human labeling, most of these data were applied in the training of the lesion detection model. We excluded images that did not contain skin lesions or that were of low resolution. This ensures the skin lesions on the images could be clearly identified. As for text, text data for training the NER model is from open datasets (He et al., 2020). Meanwhile, we selected the medical record and diagnosis image data as our training set for our classification model. The prospective dataset for testing consisted of triples (image, text, and diagnosis) of patients recruited for the study and images captured by mobile phones and texts collected by the hospital HIS system. A total of 10,406 image and text case reports were received. After applying inclusion and exclusion criteria for recruitment and checking valid text case report information that AI can recognize, 6,043 triples for 31 diseases were identified to meet the standard. The reference criteria for the diagnosis of each case are provided by dermatologists who have at least 15 years of seniority in dermatology in clinical diagnosis. Each case would be included in the dataset after image filtering and identified as the gold standard by two dermatologists simultaneously. More examples can be found in the Supplementary material.

## 4. Result

### 4.1. Overview of approach

**Figure 2:**
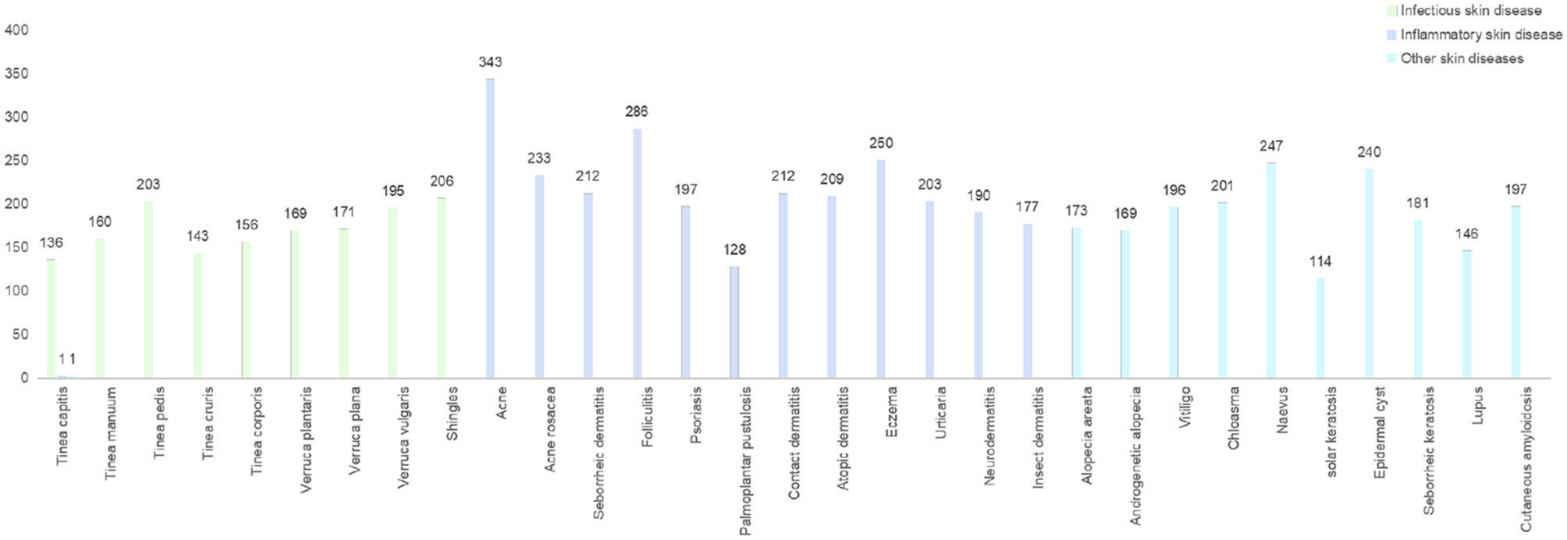
The distribution of prospective clinical cases. A dataset contained prospective clinical cases of skin diseases in China for a period of 3 months. Dermatological types were collected and assessed by outpatient dermatologists and reviewed by a panel of dermatologists.

Aiming at ethnic Chinese sources, for the first time, we collect an Asian non-cancer dataset in the form of triplets (image, text, and diagnosis). The dataset consists of over 200,000 images and 220,000 texts (He et al., 2020) from medical records, including our top collaborative hospitals and other open resources. Apart from the dataset, with a strict criterion on prospective data, we carefully selected a validation dataset including 6,043 samples from 31 diseases (Table 1, Figure 2). Using the datasets above, we propose a joint classification model based on Dual-channel Image and Extracted Text features (DIET). By using the UNet detection model, we extracted local features of the lesion. Passing the lesion and original image through a Dual-channel ResNet-based model, we captured the image features. Combining image and text features from the medical records, we generated predictive results by logistic regression. Our contributions are mainly as follows: (1) we extracted a large dataset that includes both image and text information from the medical record, which is the largest dermatological dataset for Chinese ethnic group. (2) We build a diagnosis system called DIET, which combines both image and text features based on Chinese medical records. (3) For the first time, we applied a Dual-channel image classification model on a non-cancer dermatitis dataset and utilized the UNet model (Ronneberger et al., 2015) on lesion detection.

**Table 1:** Characteristics of the prospective clinical data.

**Characteristics**	**Male, *n* (%)**	**Female, *n* (%)**	**Numbers included in study**	**Total**
Patients	3,172 (52.49)	2,871 (47.51)	35.81 (26.63)	6,043
Images	/	/	/	6,043

The dataset contained prospective clinical cases of skin diseases in China for a period of 3 months. Dermatological types were collected and assessed by outpatient dermatologists and reviewed by a panel of dermatologists.

### 4.2. Diagnosis performance findings

**Figure 3:**
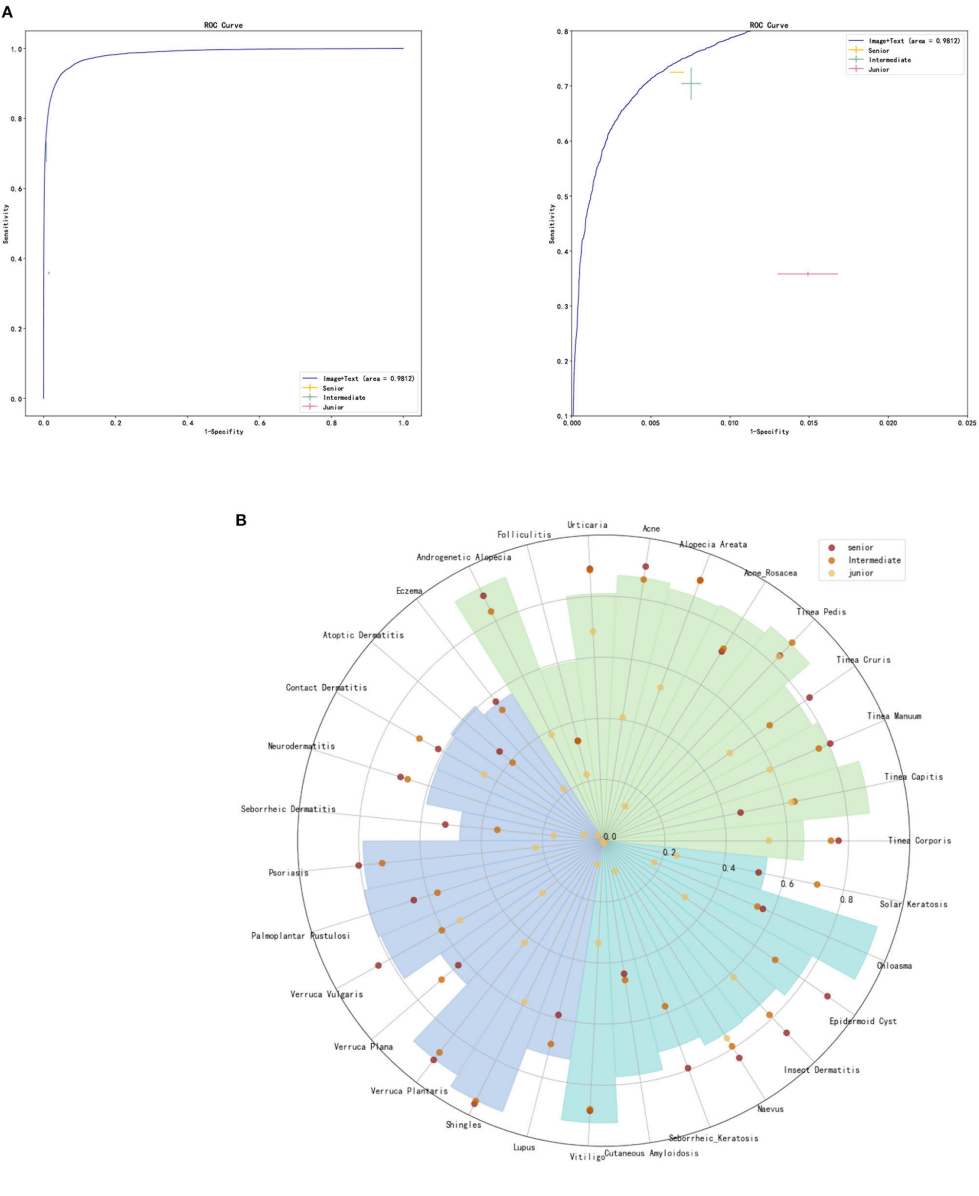
**(A)** ROC curve for DIET-AI, junior dermatologists, intermediate dermatologists, and senior dermatologists. **(B)** Skin diseases classification performance of DIET-AI and junior dermatologists, intermediate dermatologists, and senior dermatologists. The accuracy of the DIET-AI and dermatologists on clinical image + case text for all cases and 31 skin diseases on the dataset (*n* = 6,043), where the accuracies of DIET-AI and dermatologists are represented by histogram and point respectively.

Two senior dermatologists (with more than 15 years of seniority), two intermediate dermatologists (with 5–10 years of seniority) and two junior general practitioners participated in our graphical diagnostic test and completed the whole case diagnosis. The average accuracy of DIET-AI was 75.98%, 72.48% for senior doctors, 70.29% for intermediate doctors, and 35.83% for junior general practitioners. This performance was statistically non-inferior to the accuracy of DIET-AI compared to that of the intermediate doctors (*p* = 0.61), indicating that we accept the original hypothesis that DIET-AI is 5% more accurate than intermediate doctors. The receiver operating characteristic curve (ROC), the sensitivity/specificity of DIET-AI and three groups of dermatologists, are presented in Figure 3 and Supplementary Table 1. It can be seen that DIET-AI's performance in the image is 13.08% higher than that of the junior doctor group and 5.69% higher than that of the intermediate doctor group, which is similar to the senior doctor group.

### 4.3. Subgroup analysis

**Figure 4:**
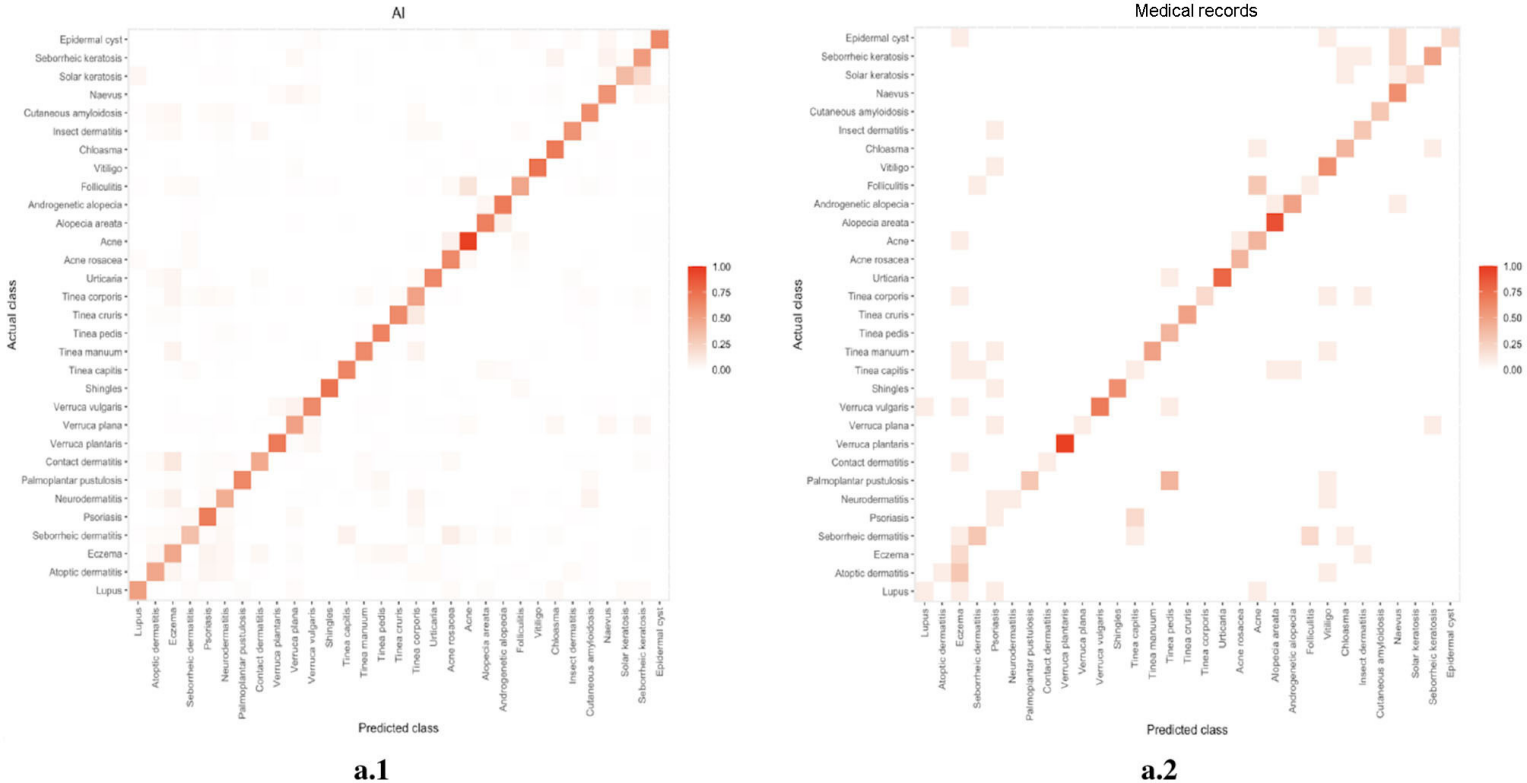
The error distribution of DIET-AI **(left)** and medical records **(right)**.

**Figure 5:**
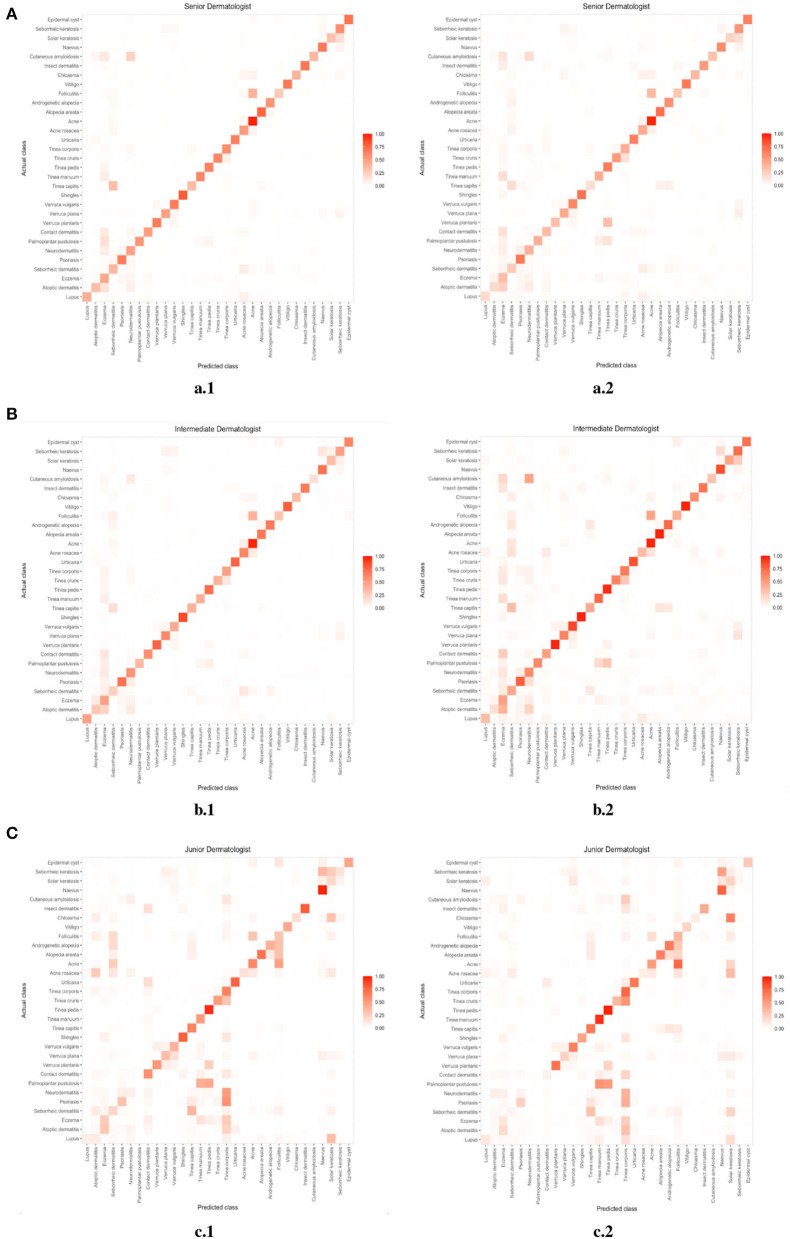
**(A)** The error distribution of senior doctors (the left image) and medical records (the right image). **(B)** The error distribution of intermediate doctors (the left image) and medical records (the right image). **(C)** The error distribution of junior doctors (the left image) and medical records (the right image).

We compared the error distribution of doctors and DIET-AI for different diseases. The confusion matrices are as in Figures 4, 5. We further calculated the diagnostic performance and consistency of DIET-AI and doctors for different diseases (Table 2). The DIET-AI showed high diagnostic capacity for inflammatory skin diseases, and its diagnostic performance converged with that of senior doctors. Besides, the performance of DIET-AI in the diagnosis of non-inflammatory skin diseases is also noteworthy. For Chloasma, where DIET-AI achieved a very high accuracy (93.71%), the diagnostic performance of senior doctor was not ideal (64.58 and 47.11%). Finally, we compared the diagnostic differences between DIET-AI and doctors on images and images with medical records. The diagnostic accuracy of DIET-AI was 45.46% for the image model and 75.98% for an image with medical records compared to 57.08% for an image and 72.48% for an image with medical records for senior doctors and 54.88% for an image and 70.29% for an image with medical records for intermediate doctors. The diagnostic accuracy of an image for junior doctors was 32.48% and an image with medical records was 35.83%. From the Appendix, we found that DIET-AI is subject to the greatest increase in accuracy for medical records, such as Lupus (increases by 49.43%), verruca plana (33.33%), insect dermatitis (28.32%), and Shingles (32.93%), while some diseases have slight fluctuations, for instance urticaria, psoriasis, and vitiligo.

**Table 2:** The Kappa coefficient compared with reference standard of 6 dermatologists and the DIET-AI.

**Dermatosis category**	**Dermatologists**			**DIET-AI**
	**Junior**	**Intermediate**	**Senior**	
**Inflammatory skin diseases with epidermal changes**				
Kappa coefficient^*^	0.201	0.629	0.664	0.681
*p*-value^†^	<0.001	<0.001	<0.001	<0.001
**Inflammatory skin diseases with dermal changes**				
Kappa coefficient^*^	0.443	0.731	0.739	0.792
*p*-value^†^	<0.001	<0.001	<0.001	<0.001
**Non-inflammatory skin diseases**				
Kappa coefficient^*^	0.283	0.666	0.698	0.743
*p*-value^†^	<0.001	<0.001	<0.001	<0.001
**Total**				
Kappa coefficient^*^	0.332	0.692	0.715	0.752
*p*-value^†^	<0.001	<0.001	<0.001	<0.001

* Compared with reference standard.

† Calculated with either the *X*^2^ test or Fisher's exact test, as appropriate.

*Compared with reference standard ^2^

Medical records helped to increase the accuracy of intermediate and senior doctors by an average of 15%. Based on the comparison of the performance of a single disease between images and images with medical records by two senior doctors, the addition of medical records resulted in a significant improvement in accuracy, with the highest improvement reaching 35.6% (tinea manus). Two senior doctors had a concurrent increase in the following diseases: lupus by 32.94 and 34.78%, respectively; Tinea capitis by 22.77 and 23.57%, respectively; Tinea corporis by 30.00 and 20.33%, respectively. However, some diseases showed a decrease in accuracy after adding medical records, as was the case for tinea pedis (9.29% decrease). Notably, for plantar warts, we observe that for a senior doctor, adding medical records leads to a drastic decrease in performance, the potential cause of which might be a lack of proficiency in certain diseases.

These five diseases were slightly or significantly increased in the intermediate doctors. For tinea manus, the two intermediate doctors increased by 10.16 and 14.33%, respectively; Tinea capitis by 41.32 and 14.84%; Tinea pedis by 5.6 and 8.13%; Lupus erythematosus by 51. 86 and 22.77%. For plantar warts, two intermediate doctors also saw a slight increase of 7.45 and 6.66%, respectively.

It is worth noting that at least one doctor for each disease showed an increase in accuracy with the addition of the medical records. Two groups of junior doctors increased by 48.39 and 61.29%, respectively, while two groups of intermediate doctors increased by 87.10 and 90.32%, and two groups of senior doctors increased by 93.55 and 96.77%. However, folliculitis was an exception. The addition of medical records decreased the accuracy to varying degrees, while DIET-AI increased by 40%. Based on interrogation information, the problems are excessively broad, and the typical diagnostic features of folliculitis are disturbed. Diagnosing based on images alone would yield some adverse information.

## 5. Discussion

We developed an artificial intelligence diagnostic tool for dermatological diseases, DIET-AI: a deep learning diagnostic model that is based on a dataset including more than 200,000 skin images and 220,000 medical records (He et al., 2020). Through relatively rigorous experiments, the model was validated on 31 common skin diseases, including the most common skin diseases. Though the selected diseases do not include skin tumors and herpetic skin diseases, which are frequently found in previous works, the prevalence of these diseases is low in the Chinese demographic and not enough data was collected in the prospective dataset's validation set. These two groups of diseases are critical in the clinical diagnostic process and will be added to the model in the future. Our experiments show that DIET-AI's performance on common skin diseases is comparable to that of senior doctors. According to our hypothesis testing results, DIET-AI outperformed intermediate doctors by 5% and was comparable to the senior doctors. As for image-only case, based on the current data, the DIET-AI image performance is higher than the performance of junior doctors, and slightly lower than the intermediate and senior doctors, partly due to DIET-AI's interpretation errors caused by the environment and image quality. We will further reduce the noise from the image context, continue to collect high-quality data, wash low-quality data, improve the robustness of the image model, and build a large library of high-quality dermatology images. Thus, a more effective image model will also improve the total performance of DIET-AI.

Specifically for fine-grained diseases, DIET-AI is better at dermal inflammatory skin diseases: 9 out of 11 diseases saw a performance of about 80%. For folliculitis, which has an unsatisfactory performance, the doctors also do not have an ideal performance. Meanwhile, DIET-AI has higher diagnostic performance than physicians in this group of diseases, and the diagnostic performance of DIET-AI converges with that of senior doctors. Our model does not perform ideally on epidermal inflammatory skin diseases: the performance of eczema, atopic dermatitis, contact dermatitis, and neurodermatitis are only at about 60% with medical records, while for seborrheic dermatitis and verruca plana, the accuracy cannot reach 50%. This finding is similar to that of physicians and clinical perceptions, and further clarification of diagnostic criteria for atopic dermatitis, eczema, neurodermatitis, and contact dermatitis is needed in the future. The image diagnosis is effective for non-inflammatory skin diseases in general, for vitiligo for instance, the difference between the DIET- AI diagram and the textual diagnosis was only 2.35%, and it achieved an image performance of 90%. For inflammatory skin diseases, DIET-AI and doctors tend to combine image and text to give a diagnosis, such as acne rosacea and lupus, which need more medical records, and it is more suitable to try an AI diagnostic model with image and text based on our prospective research. Intuitively, the performance of DIET-AI should be enhanced based on the medical records, which provide more information, leading to better model generalization. This joint information makes DIET-AI robust under diverse scenarios. From our experiments, we find that intermediate doctors rely more on medical records than senior doctors, which is also in line with our clinical research findings. Therefore, it is necessary for DIET-AI to provide text and for doctors to not rely on the image to make direct judgements. Our research has further clarified the importance of consultation information. However, to compare the diagnostic performance of doctors and DIET-AI, this information needs to be used in a more clinical setting. The current consultation sessions take a long time on average to collect sufficient diagnostic consultation information and are not yet able to assist dermatologists well in clinical scenarios. In the future, we will independently develop an AI consultation system that can be used in scenarios such as when teaching primary dermatologists to standardize consultations, for pre-consultation of e-consulting and pre-visits, and integrating case information to help disease management and clinical research. This will also save doctors' consultation time and allow them to spend more time on patient treatment and humanistic care, which cannot be replaced by artificial intelligence (Stead, 2018).

Finally, in the empirical study, DIET-AI shows its potential in real-world clinical applications due to its effectiveness under diverse scenarios. We prospectively conducted the collection of data from a wide variety of regions, medical centers, and even mobile devices. During this collection, the performance of DIET-AI remains relatively stable, which demonstrated the robustness of our method. The comprehensive diagnostic performance of DIET-AI on 31 common diseases was similar to the level of senior dermatologists, comparable to intermediate dermatologists, and significantly better than primary general practitioners. In our prospective study, we found that treatment resources were uneven, indicating the diagnostic gap is large between doctors from top hospitals and primary hospitals but small among doctors of different types of seniority from the same tier of hospitals. Our analysis has not fully captured this imbalance among treatment resources. Meanwhile, apart from images and text, other hardware in hospitals might also improve the diagnostic performance of doctors, which we will introduce to our future work. This research provides ideas for future exploration of scenarios in which the performance of AI can be assessed more objectively and realistically. With the development of information extraction technology, we are bound to get more and higher-modal data that will not only provide personal diagnosis and assist coaching AI but also better match the real clinical scenarios of doctors, thus better comparing the performance of doctors and AI and clarifying the scenarios in which AI can be used.

## Data availability statement

The original contributions presented in the study are included in the article/Supplementary material, further inquiries can be directed to the corresponding authors.

## Ethics statement

The studies involving human participants were reviewed and approved by the Ethics Committee of the Third Affiliated Hospital of Chongqing Medical University [2022 Research Ethics Approval No. 44]. Written informed consent was obtained from the participants or their legal guardians/next of kin for participation in the study and for the publication of any potentially identifiable images/data included in the manuscript.

## Author contributions

HL, PZ, WZ, and JL initiated the project and provided guidance on the concept and design. PZ, XL, SY, and YZ wrote the manuscript. HC, KD, YF, XH, GH, XJ, LL, TQ, TS, FY, and DZ provided clinical expertise, guidance on the study design, and collected and analyzed the data. ZW and KH developed the network architecture and data, modeling infrastructure, and training and testing setup. YT and XX created the figures, wrote the methods, and performed additional analysis. HL, GZ, JL, and DZ supervised the project. All authors discussed the results and reviewed the manuscript. All authors contributed to the article and approved the submitted version.

## Footnotes

^1^ https://github.com/LeeSureman/Flat-Lattice-Transformer

^2^The reference criteria for the diagnosis of each case are provided by dermatologists who have at least 15 years of seniority in dermatology in clinical diagnosis.
