# Supplementary material for: Deep skin diseases diagnostic system with Dual-channel Image and Extracted Text
Source: Front Artif Intell. 2023 Oct 19;6:1213620. doi: 10.3389/frai.2023.1213620 (PMC10620802; doi:10.3389/frai.2023.1213620)
Supplement: Supplementary file 1 [file Data_Sheet_1.PDF]

Supplementary

1 EXAMPLES OF THE DATA TRIPLES

Deseases	Images	Texts
Lupus	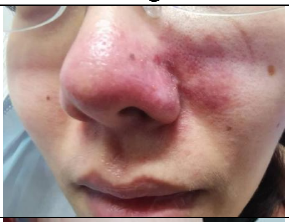	Female, 30 years old. I've observed a red patch on my nose and cheeks, which is also slightly itchy. My mother has previously exhibited similar red patch on her face.
Eczema	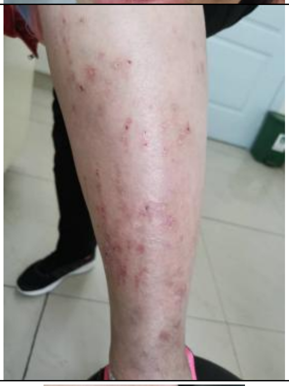	Male,22 years old. About a month ago, I began to notice small red rashes on my legs, which can be particularly itchy at times. The skin on my legs is also very dry.
Cutaneous amyloidosis	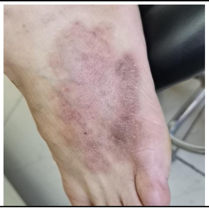	Male,35 years old. Initially, there were pin-sized brown papules on the dorsum of my foot, which felt very itchy. I constantly felt the urge to scratch them, and over time, the area expanded, with the skin on my foot becoming notably rougher.

Table S1. Three examples of the data triples. Notice that the red marked part is the extracted name entity from text with medical information.

2 THE FULL LIST OF AI AND DERMATOLOGIST PERFORMANCES

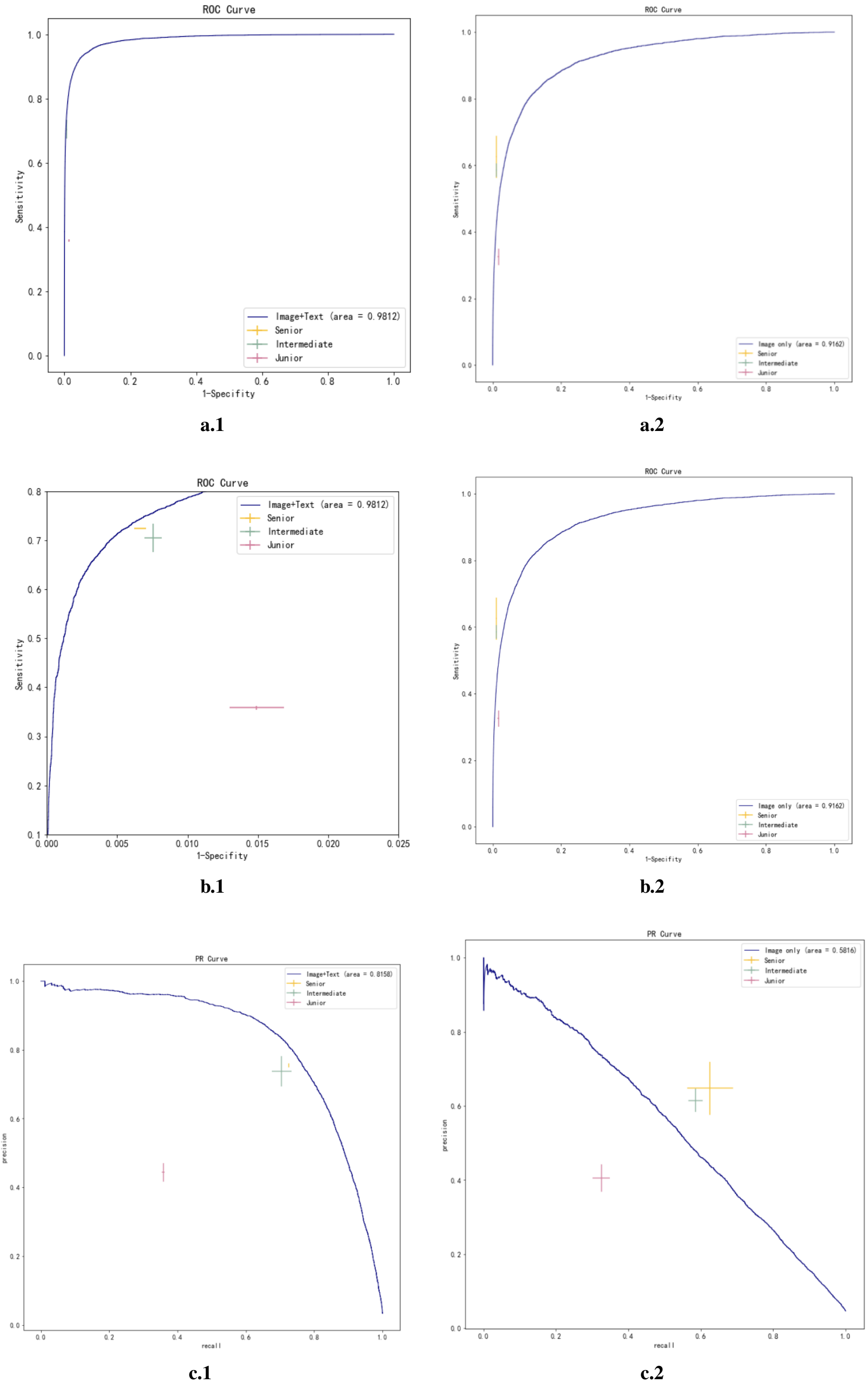

Figure S1: Skin diseases classification performance of the DIET-AI and junior dermatologists, intermediate dermatologists and senior dermatologists.

In the Figure S1, a.1 and a.2 shows that the performance of DIET-AI is slightly higher than senior and intermediate, and much higher than junior dermatologists in the classification of skin diseases with clinical images + medical records as input. Meanwhile, the DIET-AI is superior to junior dermatologists in the classification of skin diseases with only clinical images as input. Our DIET-AI has been tested against at least 6 dermatologists in 31 dermatological identifications. Performance is measured by sensitivity, true positive rate, specificity, and true negative rate. The dermatologists output a prediction for each image, and the crosses represent the average of each group of dermatologists. AUC is a performance measure for DIET-AI with a maximum value of 1. If the dermatologist's sensitivity-specificity point lies below the ROC curve, the DIET-AI outperforms the dermatologist in most cases. b.1 and b.2 are the magnified edition of a.1 and a.2. Likewise, we draw the P-R curve (c.1 and c.2), of which upper-right direction implies a higher performance.
